# Effect of clinician information sessions on diagnostic testing for Chagas disease

**DOI:** 10.1371/journal.pntd.0010524

**Published:** 2022-06-16

**Authors:** Helen Mahoney West, Carly E. Milliren, Jennifer Manne-Goehler, Jillian Davis, Jaime Gallegos, Juan Huanuco Perez, Julia R. Köhler

**Affiliations:** 1 Division of Infectious Disease Boston Children’s Hospital, Boston, Massachusetts, United States of America; 2 Institutional Centers for Clinical and Translational Research, Boston Children’s Hospital, Boston, Massachusetts, United States of America; 3 Massachusetts General Brigham Hospital, Boston, Massachusetts, United States of America; 4 East Boston Neighborhood Health Center, Boston, Massachusetts, United States of America; 5 Department of Pediatrics, Harvard Medical School, Boston, Massachusetts, United States of America; Federal University of Ceará, Fortaleza, Brazil, BRAZIL

## Abstract

**Background:**

Chagas disease is a potentially life-threatening neglected disease of poverty that is endemic in continental Latin America. Caused by *Trypanosoma cruzi (T*. *cruzi)*, it is one of six parasitic diseases in the United States targeted by the Centers for Disease Control as a public health problem in need of action. An estimated 300,000 people are infected with *T*. *cruzi* in the United States (US). Although its morbidity, mortality and economic burden are high, awareness of Chagas disease is lacking among many healthcare providers in the US. The purpose of this analysis is to determine if the number of diagnostic tests performed at a community health center serving an at-risk population for Chagas disease increased after information sessions. A secondary aim was to determine if there was a difference by provider type, i.e., nurse practitioner vs. physician, or by specialty in the number of patients screened.

**Methodology/Principal findings:**

We conducted a retrospective data analysis of the number of Chagas serology tests performed at a community health center before and after information sessions for clinicians. A time series analysis was conducted focusing on the Adult and Family Medicine Departments at East Boston Neighborhood Health Center (EBNHC). Across all departments there were 1,957 *T*. *cruzi* tests performed before the sessions vs. 2,623 after the sessions. Interrupted time series analysis across departments indicated that testing volume was stable over time prior to the sessions (pre-period slope = +4.1 per month; p = 0.12), followed by an immediate shift after the session (+51.6; p = 0.03), while testing volume remained stable over time after the session (post-period slope = -6.0 per month; p = 0.11).

**Conclusion/Significance:**

In this study, Chagas testing increased after information sessions. Clinicians who began testing their patients for Chagas disease after learning of the importance of this intervention added an extra, potentially time-consuming task to their already busy workdays without external incentives or recognition.

## Author summary

Chagas disease is a potentially fatal neglected disease of poverty. It is endemic in continental Latin America with an estimated 300,000 cases in the United States, primarily among low-income people who have immigrated to the US from Latin America. Few Chagas screening programs have been established in the US. Existing recommendations for Chagas disease testing and treatment are rarely followed for many reasons including a paucity of knowledge among providers. We aimed to determine if the number of Chagas tests performed increased after information sessions at a community health center. A secondary aim was to determine if there was a difference in number of tests performed by provider type. We found that the number of *T*. *cruzi* serologies performed in the ten months after information sessions increased significantly over that in the ten preceding months. Chagas testing increased across departments, though Chagas diagnostics were an extra and unmitigated time burden on clinicians. Increasing provider knowledge is a major step to increase diagnosis and treatment of this neglected disease, when clinicians are motivated by their inherent prosocial preferences including altruism i.e., by the positive impact of their work on patients’ lives.

## Introduction

Chagas disease is one of six parasitic diseases in the United States targeted by the CDC as a public health problem in need of action [1]. It is a potentially life-threatening insect-borne disease caused by the parasite *Trypanosoma cruzi*. The insect vector inhabits cracks in walls and roofs of houses made of natural materials like thatched roofs and adobe walls. In endemic regions, Chagas disease is usually acquired in childhood and is strongly associated with poverty [2]. Unless treated, infection is life-long. An acute phase follows initial infection, which may present with influenza-like symptoms and, rarely, acute myocarditis or meningoencephalitis [3,4]. The chronic phase is asymptomatic unless heart or gastrointestinal disease manifest, hence diagnosing asymptomatic individuals is critical [4–6]. Mother-to-child transmission can occur outside endemic regions in an estimated 1–10% of births among infected mothers [7]. Congenital infection can be prevented by antiparasitic therapy before pregnancy [6–9].

After acute infection, if untreated, an estimated 20–30% of infected individuals will go on to relentlessly progressive heart disease including cardiomyopathy and arrhythmias and 10% to gastrointestinal disease (primarily megaesophagus or megacolon) [4,5]. An estimated 6–8 million people are infected with *T*. *cruzi* in Latin America [10,11], while an estimated 300,000 are living with Chagas disease in the United State [12]. In the US, an estimated 30,000–45,000 people live with undiagnosed Chagas cardiomyopathy and an estimated 63–300 cases of congenital infection occur per year [13]. A recent study looking at the prevalence of Chagas disease among the Latin American-born population in Los Angeles reported a prevalence of 1.24% or more than 30,000 individuals in Los Angeles County infected with Chagas disease [14]. Globally, it is estimated that only 1% of infected individuals receive treatment [15]. The economic burden of Chagas disease is considerable with global healthcare costs estimated to be $7–19 billion annually in 2012 dollars [16]. The US and Canada account for more than 10% of these costs [16]. In the US, a lack of awareness of Chagas disease by medical and public health professionals has been documented [17–21]. Outside of blood and organ donor screening programs, few efforts to detect and address the disease exist within the US healthcare system, even though screening for other latent infections like tuberculosis and syphilis is routine.

The World Health Organization has developed guidelines for the diagnosis and treatment of Chagas disease [22]. But the guidelines are rarely followed due to a variety of factors, including low provider awareness. Multiple surveys of US physicians found a paucity of knowledge of Chagas disease across disciplines [17–21]. In one study, 47% of obstetricians and 23% of cardiologists reported no knowledge of Chagas disease while approximately half of these specialists reported a lack of confidence that their knowledge was up to date [17]. Studies among pediatricians and obstetrician-gynecologists (OBGYNs) identified limited awareness of the risk of congenital transmission [18,19]. A recent survey of OBGYN and Family Medicine providers in a clinical department that had received an information session, found that although the majority of respondents reported being familiar with Chagas disease only one third knew how to order a test [20]. In a survey of Appalachian Ohio physicians 80% of respondents reported “limited” or “very limited” knowledge Chagas disease [21]. These studies underscore the need for increased Chagas disease education among clinicians as a means of improving the identification, diagnosis, and treatment of at-risk populations. To improve health outcomes, interactive education involving multidisciplinary healthcare providers, in conjunction with implementation of guidelines has been recommended [23].

The primary aim of this study was to determine if the number of Chagas tests performed at a large community health center in Boston, Massachusetts increased after information sessions. A secondary study aim was to determine if there is a difference by provider type in the number of patients tested. Identifying variation in approaches to Chagas disease testing between nurse practitioners (NP’s) and physicians may help focus educational efforts.

## Methods

### Ethics statement

This study was approved with exempt status by the Institutional Review Board at Boston Children’s Hospital (IRB Protocol # IRB-P00032812). This is a retrospective analysis of de-identified data.

### Setting

We analyzed data from East Boston Neighborhood Health Center in East Boston, Massachusetts. EBNHC serves a neighborhood of approximately 50,000 residents. 56% of East Boston residents are of Latin American origin, 80% of whom are from Central and South America, the population at risk for Chagas disease. EBNHC is also used by residents of adjacent towns and neighborhoods [24]. EBNHC is one of the largest federally qualified community health centers in the US with an estimated 300,000 patient care visits per year and an estimated 28,000 patients seen in the Adult Medicine clinic over a 4-month period. EBNHC employs over 50 healthcare practitioners providing primary care services in Pediatrics, Women’s Health, Adult and Family Medicine as well as Obstetrics–Gynecology (OB/GYN) services and care for children with special health care needs. EBNHC serves a significant number of patients who have themselves migrated or were born to a mother who migrated to the US from Latin America. As a community health center, it serves mostly low-income patients, and hence is expected to care for a substantial number of undiagnosed Chagas disease patients. Results of Chagas diagnostics at EBNHC according to demographics and country of origin are currently being analyzed and will be reported separately.

### Information sessions

Multiple Chagas disease information sessions were offered to providers in 2015–2019 at EBNHC. Information sessions were between 20 minutes and one hour long and were conducted by two members of the “Strong Hearts” project, J.M.G. and J.R.K. who had or were in the process of obtaining Infectious Disease subspecialty training. The two sessions analyzed for this report were held by a single Strong Hearts member, J.R.K., in two departments on the same day, 5/15/2018; hence content- or stylistic differences between these sessions were likely to be small. In addition to information about the transmission, epidemiology, and clinical course of Chagas disease, as well as diagnosis and treatment, sessions stated the estimated prevalence of the disease in the United States and the fact that less than 1% of people living with this disease are treated in the US.

Other aspects of the neglect of this disease were highlighted: the poor sensitivity and specificity of available diagnostic tests, the fact that current antiparasitic medications for Chagas disease were developed in the 1960’s and 1970s, new regimes are not of commercial interest for the pharmaceutical industry and hence are primarily being developed by the Drugs for Neglected Diseases Initiative, and that medical providers and at-risk communities are often unaware of the disease. We described the vicious cycle how unawareness by medical providers of the disease leads to lack of efforts to diagnose it, and how low volumes of diagnostic testing leads to disinterest by commercial laboratories to offer improved diagnostic options.

It was stated that globally the disease is neglected because the people it affects are neglected and devalued. Slides from information sessions as well as the dates of the prior sessions can be found at https://strongheartsma.wixsite.com/chagas/provider-faqs.

### Testing guidelines

Among EBNHC patients those at risk for Chagas disease were defined as having ever resided in a country of continental Latin America (excluding the islands of the Caribbean), all of which are endemic for Chagas disease, or being born to a mother who resided in Latin America for 6 months or more.

In the time period covered by this study, an additional criterion for testing was age <50 years, given general recommendations at the time that generally limited antiparasitic therapy to this age range. This criterion has since been revised with the recognition that diagnosing older patients of any age is important to optimize their cardiologic care, which differs from that of patients with other heart diseases [25].

Serological diagnosis of Chagas disease requires positive results in two independent tests with distinct methodologies, because there is currently no single diagnostic test with sufficient sensitivity and specificity [26]. For this reason, to make a diagnosis of Chagas disease under current conditions in the US, an initial positive serologic test performed by a commercial laboratory must be confirmed by the Centers for Disease Control and Prevention (CDC) parasitology laboratory. Send outs to CDC laboratories are routed through each state’s Public Health department and require submission of a special CDC requisition form. To make a diagnosis of Chagas disease for one patient, two serum samples therefore must be submitted sequentially to two different organizations using different requisition systems. Results faxed from the CDC must be scanned into electronic medical record systems and specifically noted by a clinician.

Patients in whom Chagas disease was diagnosed in this way were referred for evaluation and treatment to the tertiary referral center serving EBNHC. Clinicians were aware that testing had therapeutic and management consequences for their patients.

After initial information sessions were conducted in each of the clinical departments of the health center in 2015–2016, a plan for diagnosing at-risk patients was developed by Strong Hearts volunteers J.H.P. and J.R.K. and approved by the board of the health center in December 2016. After another information session on March 21, 2017 in the EBNHC Adult Medicine Department, clinicians in this department began testing their patients for Chagas disease. The intention was to test every patient with a Latin American country of origin and testing was and is based on each individual clinician’s discretion. Chagas serology can be ordered within the commercial laboratory system (Quest Diagnostics) serving the health center. During the study period, Quest Diagnostics used the Hemagen ELISA as the diagnostic assay for *T*. *cruzi* serology [27]. The EBNHC on-site laboratory, a Quest satellite, divides serum samples submitted for Chagas testing and retains an aliquot until the results of the ELISA become available from the central Quest facility. Strong Hearts volunteers follow up on positive ELISA test results and ensure the saved aliquot is sent for confirmatory testing to the CDC. When a positive confirmatory result is received from the CDC, volunteers notify the patient’s primary care physician and the patient and enter a referral for an Infectious Disease clinic visit into the electronic medical record.

De-identified Chagas testing data are tracked by “Strong Hearts” volunteers. The data include patient age and gender, patient’s country of origin, date of screening test, initial results, and CDC confirmatory test results.

### Data collection

We retrospectively extracted the number of *T*. *cruzi* serology samples sent at EBNHC between June 1, 2017 and March 31, 2019 for two departments (Adult Medicine and Family Medicine) from the repository data base. Both departments received a Chagas information session in May 2018. For the purposes of this analysis, Chagas tests prior to May 2018 through the beginning of the study period, 6/1/2017–5/14/2018, are considered ‘before’ the session and those in May 2018 through the end of the study period, 5/16/2018–3/31/2019, are considered ‘after’ the session. While nine information sessions were conducted at EBNHC between 2015 and 2019, the two sessions held on the same day, May 15, 2018 were selected for inclusion in this analysis. This time point was chosen to minimize confounding by the development of the testing system over the year prior to these sessions.

We were unable to ascertain the total number of patients at risk for Chagas disease seen in each time period as we did not have access to countries of origin for all patients seen in these departments. For this reason, we use the total number of patient visits in each department as a proxy for the number of at-risk patients.

### Statistical analysis

We report mean (standard deviation) for continuous variables and frequency (percent) for categorical variables. We compared time periods before and after information sessions on demographic characteristics (age, gender, country of origin), initial test results and CDC confirmatory test results, department and provider type using t-tests for continuous variables (age) and chi-square tests for categorical variables.

To examine the trend in monthly Chagas testing volume before and after information sessions, we used interrupted time series regression analysis. We examined the trend in volume overall as well as stratified by department and by provider type (physicians, mid-level providers which included nurse practitioners and physician assistants). Models tested for a change in monthly volume over time prior to the session (pre-session slope), an immediate shift (post-session intercept), and a shift in the change over time after the session (post-session slope). If applicable, non-significant terms (p>0.2) for the change over time (pre or post) were removed to construct a parsimonious model.

While unable to ascertain the number of at-risk patients screened during each time period, we examined the total patient visit volume to ensure a change in the number of tests performed was not due to a change in total visit volume. The trend in total monthly visit volume across and within departments was examined using interrupted time series regression analysis testing for a change over time prior to the session, an immediate shift following the session, and a shift in the change over time in the period after the session. We also examined the overall trend in monthly testing volume normalizing to the total patient visits to calculate testing volume per 1,000 patient visits.

All analyses were performed in SAS (v9.4; Cary, NC) at an alpha-level of 0.05.

A de-identified dataset is stored in a repository on Open Science Framework and available at the following link: https://osf.io/cw8hn/?view_only=8ecbf34e672d46649d890f414ae0bd99.

## Results

We retrospectively analyzed the number of *T*. *cruzi* serology samples sent at EBNHC between June 1, 2017 and March 31, 2019 for two departments (Adult Medicine and Family Medicine) from a repository data base. Both departments received a Chagas information session in May 2018. Across both departments, there were a total of 4,580 *T*. *cruzi* serology tests performed in the twenty-two-month study period; 1,957 (43%) prior to information sessions and 2,623 (57%) following the sessions. **Table 1** includes demographics and screening results for the periods before and after information sessions. The majority of screened patients were female with an average age of 35 years. Most patients were from Central and South America with El Salvador and Colombia the most common countries of origin accounting for 68% of screening tests. There was no difference before and after sessions with respect to age, gender, or region of origin.

**Table 1:** Demographics, country of origin and test results for patients tested for Chagas disease before and after information sessions (N = 4,580).

	Tests Performed, *n (%)*		p-value
	Before (n = 1,957)	After (n = 2,623)	
**Age** (years), *mean (SD)*	35.1 (10.8)	35.0 (10.7)	0.60
**Female Gender**	1,259 (64%)	1,639 (62%)	0.20
**Region of Origin**			0.48
Central America	1,263 (65%)	1,703 (65%)	
South America	611 (31%)	796 (30%)	
Caribbean	32 (2%)	44 (2%)	
United States	28 (1%)	48 (2%)	
Africa	11 (1%)	23 (1%)	
Other^a^	1 (<1%)	2 (<1%)	
Unknown			
**Commercial Lab Screening Test Result**			0.10
Negative	1,854 (95%)	2,512 (96%)	
Positive	85 (4%)	99 (4%)	
Indeterminate^b^	18 (1%)	12 (<1%)	
**CDC Confirmatory Test Result** (n = 164)^c^			0.16
Negative	59 (77%)	58 (67%)	
Positive	18 (23%)	29 (33%)	

a Includes n = 1 Europe and n = 2 Asia

b Indeterminate–commercial laboratory (Quest Diagnostics) description of ELISA results according to their standards

c Excludes n = 10 confirmatory results that were not yet available (pending) at the time of data collection and n = 10 confirmatory tests not sent due to patient age.

Most screening tests at Quest Diagnostics were negative while 184 (4%) were found to be positive and 30 (<1%) were indeterminate. There was no difference in the screening test result before and after the information sessions (p = 0.10). Of the 164 tests sent to the CDC for confirmatory testing (i.e., sent to the CDC if initial ELISA test was positive or indeterminate) 47 (29%) resulted positive, and 117 (71%) resulted negative. Results of CDC confirmatory testing were similar before and after the sessions (p = 0.16) (Table 1).

**Fig 1:**
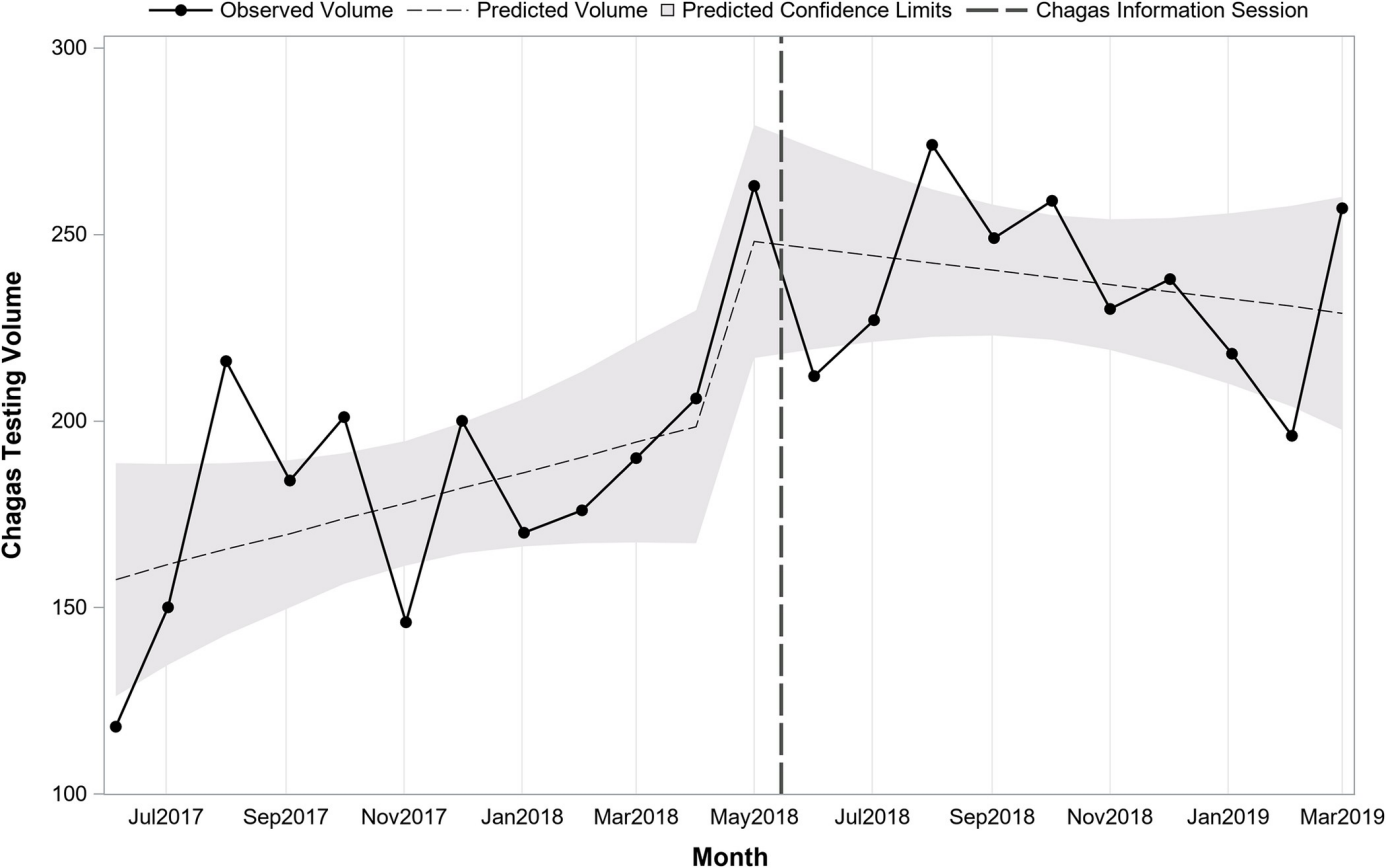
Overall trend in monthly Chagas screening test volume before and after Chagas information sessions (N = 4,580 tests; 22 months).

**Fig 2:**
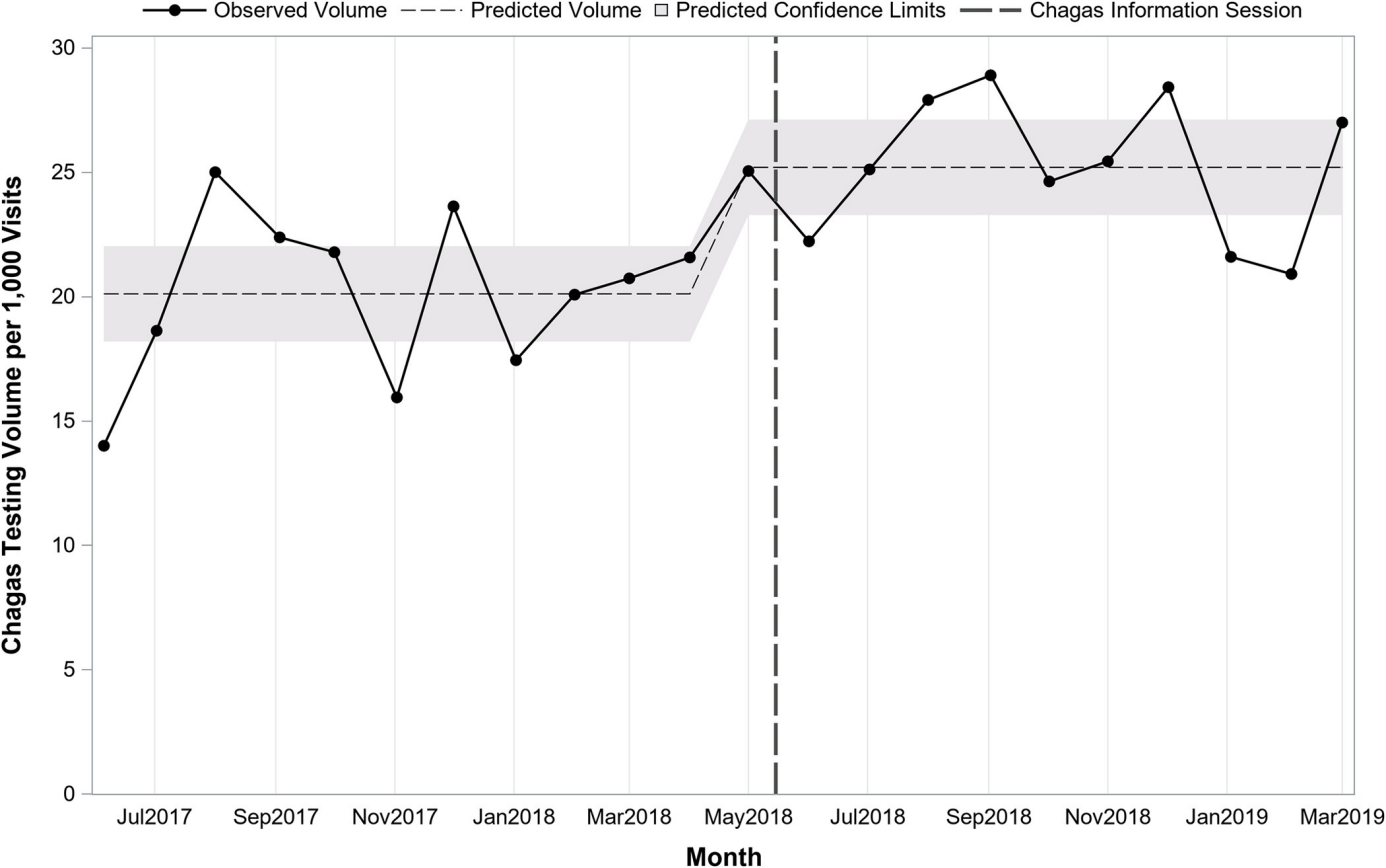
Overall trend in monthly Chagas screening test volume per 1,000 patient visits before and after Chagas information sessions (N = 201,865 patient visits; 4,580 tests; 22 months).

Interrupted time series analysis indicated that prior to the sessions at the start of the study period in May 2017, there was a predicted average of 157 tests monthly (pre-period intercept) and testing volume was increasing slightly over time by 4.1 per month (pre-period slope; p = 0.12). Immediately following the information sessions in May 2018, there was a significant increase in the number of tests (+51.6; p = 0.03). After the sessions, the change in volume over time was stable, decreasing slightly but not significantly by 6.0 per month (post-period slope; p = 0.11). **Fig 1** presents monthly testing volume overall before and after the information sessions on 5/15/2018. **Fig 2** presents overall monthly testing volume per 1,000 patient visits before and after the information sessions on 5/15/2018. Interrupted time series analysis indicated that prior to the sessions there was an average of 20.1 tests per 1,000 patient visits. Following the sessions, there was an immediate increase of 5.1 (p<0.001) for a total average of 25.2 per 1,000 patient visits.

**Fig 3:**
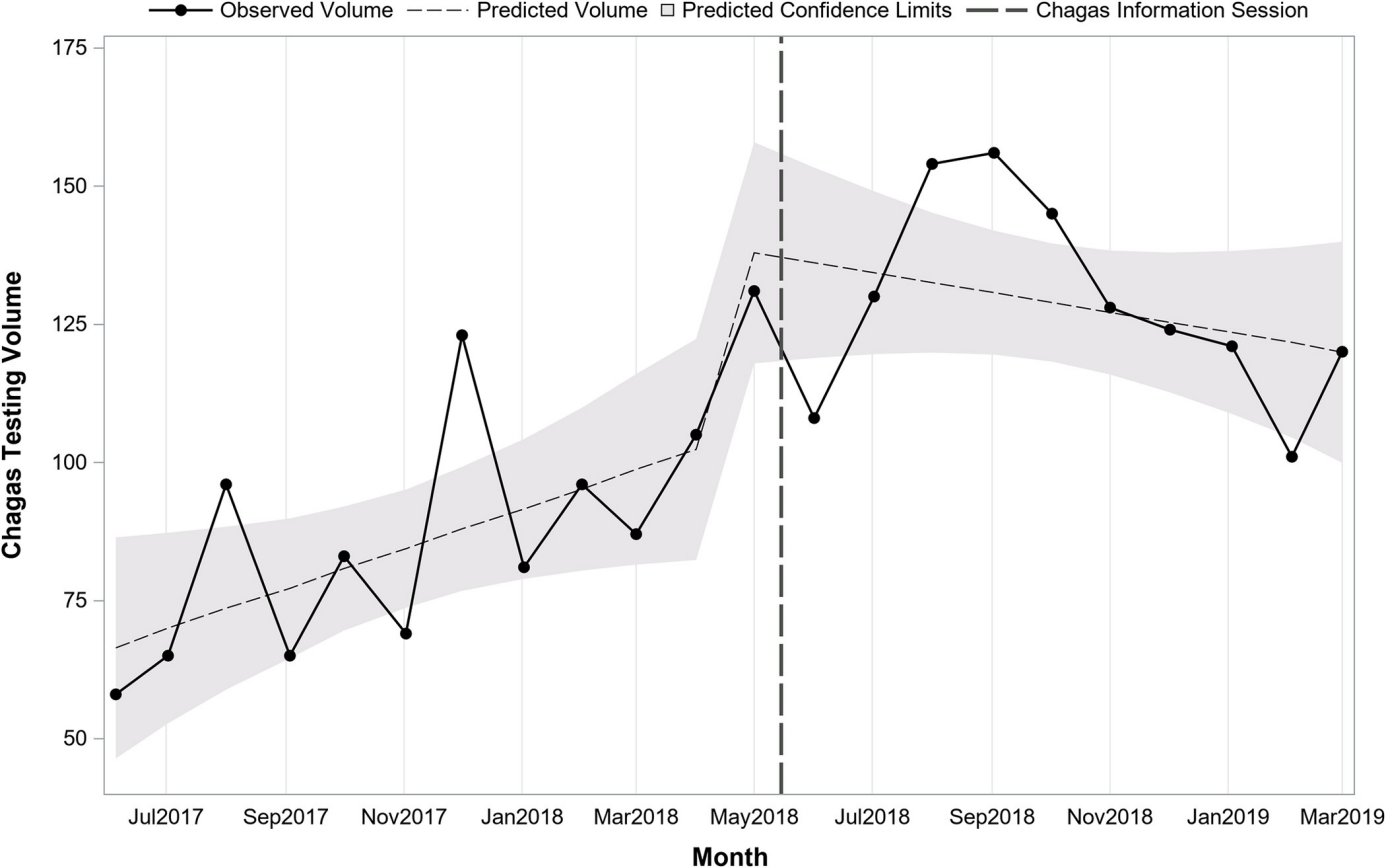
Trend in monthly Chagas screening test volume before and after Chagas information session in Family Medicine (n = 2,346 tests; 22 months).

**Fig 4:**
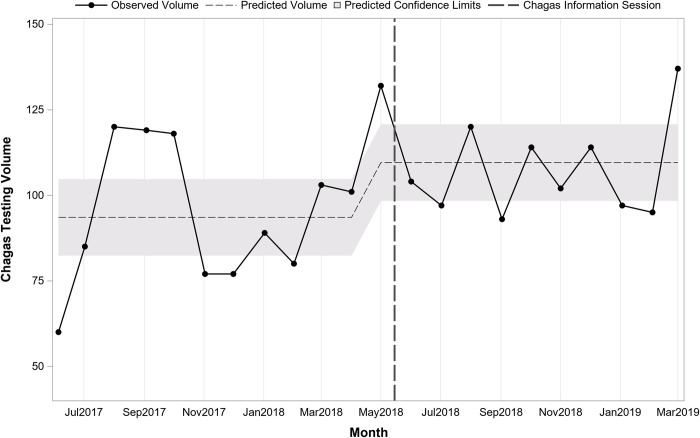
Trend in monthly Chagas screening test volume before and after Chagas information session in Adult Medicine (n = 2,234 tests; 22 months).

**Table 2** presents testing in the periods before and after information sessions by department and provider type. Testing before and after the sessions differed significantly by department (p<0.001) with Adult Medicine accounting for a larger proportion of tests prior to the sessions and Family Medicine accounting for a larger proportion after the sessions. Interrupted time series regression analysis stratified by department indicated monthly testing volume in Family Medicine (**Fig 3**) was similar to the overall findings while for Adult Medicine (**Fig 4**) there was a significant shift following the session. In Family Medicine, testing volume was increasing over time prior to the session (pre-session slope = +3.6; p = 0.04). Immediately following the session, there was an increase of 37.4 (post-session intercept; p = 0.02) followed by a slight decline over time by 5.4 per month (post-session slope; p = 0.03). In Adult Medicine, a model testing just for a shift indicated that there was an immediate increase of 16.0 (p = 0.047) following the session.

**Table 2:** Chagas testing before and after information sessions by department and provider type (N = 4,580).

	Tests Performed, n (%)		p-value
	Before (n = 1,957)	After (n = 2,623)	
**Department**			<0.001
Adult Medicine	1,029 (53%)	1,205 (46%)	
Family Medicine	928 (47%)	1,418 (54%)	
**Provider Type**			<0.001
Nurse Practitioner (NP)	1,041 (53%)	1,254 (48%)	
Physician (MD, DO)	834 (43%)	1,268 (48%)	
Physician Assistant (PA)	82 (5%)	101 (4%)	

**Fig 5:**
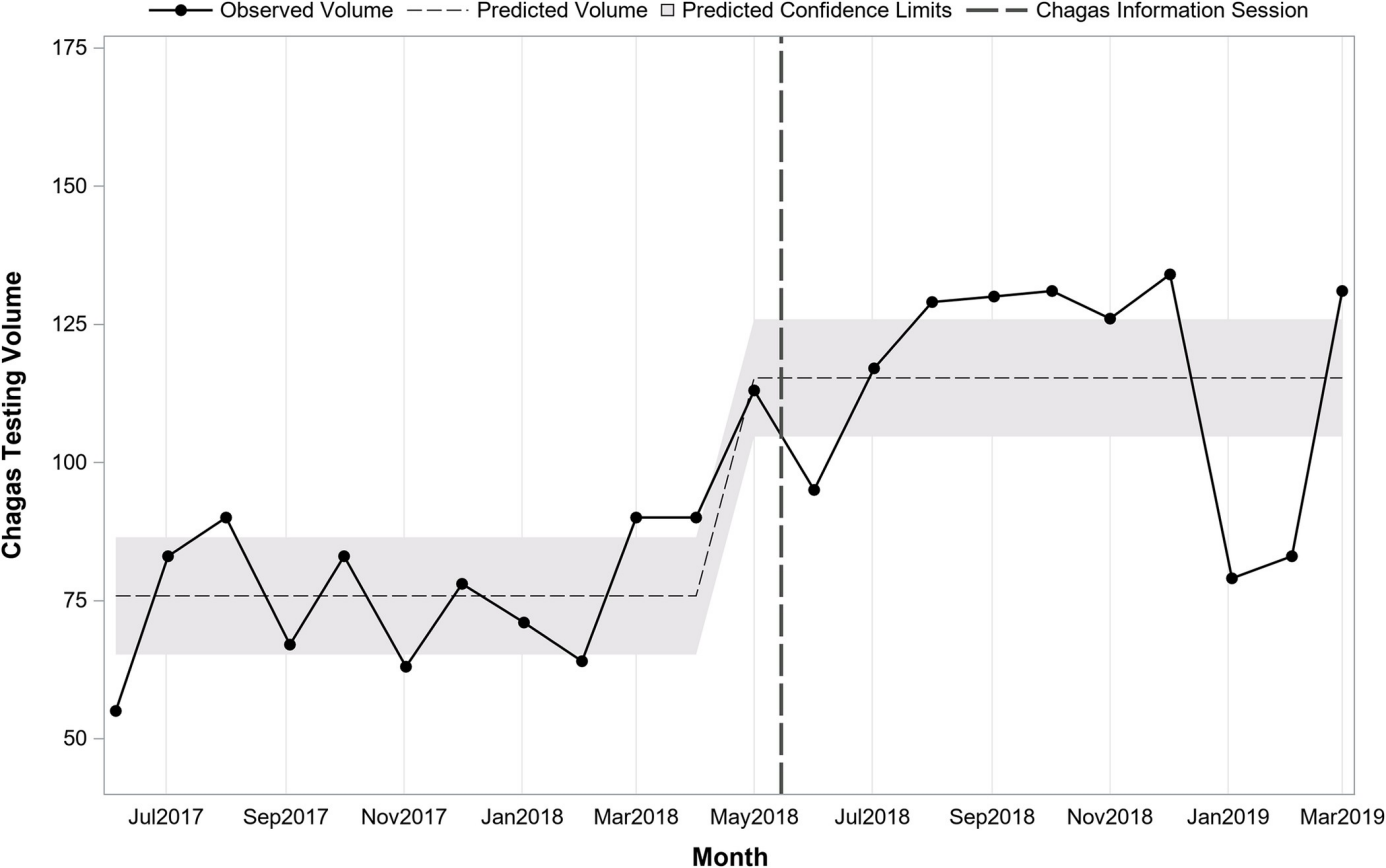
Trend in monthly Chagas screening test volume before and after Chagas information sessions for physicians (n = 2,102 tests; 22 months).

**Fig 6:**
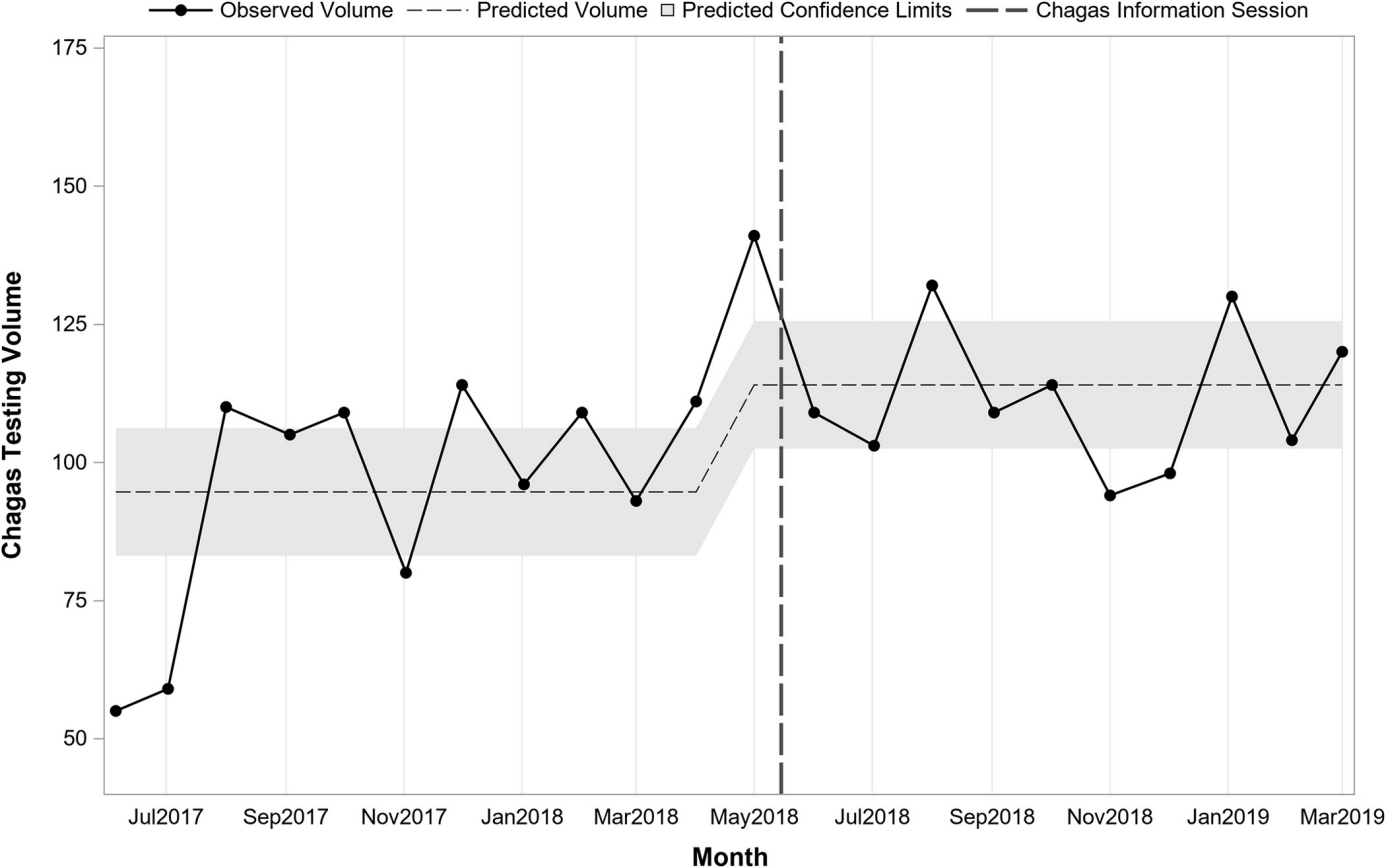
Trend in monthly Chagas screening test volume before and after Chagas information sessions for mid-level providers (nurse practitioners or physician assistants) (n = 2,478 tests; 22 months).

Testing before and after the sessions differed significantly by provider type (p<0.001; Table 2). Prior to the sessions, the majority of tests (53%) were ordered by nurse practitioners while 43% were ordered by physicians. After the sessions, nurse practitioners and physicians ordered an equal proportion of tests (both 48%). Interrupted time series analysis stratified by provider type indicated an immediate shift following the sessions for both physicians (**Fig 5**) and mid-level providers (NP or PA; **Fig 6**). For physicians, a model assessing only the post-sessions shift indicated that physicians ordered an average of 75.8 tests per month prior to the sessions followed by an immediate increase of 39.5 after the sessions (p<0.001). Prior to the sessions, mid-level providers ordered an average of 94.6 tests per month. Following the sessions, there was an immediate increase of 19.4 (post-session intercept; p = 0.02).

**Fig 7:**
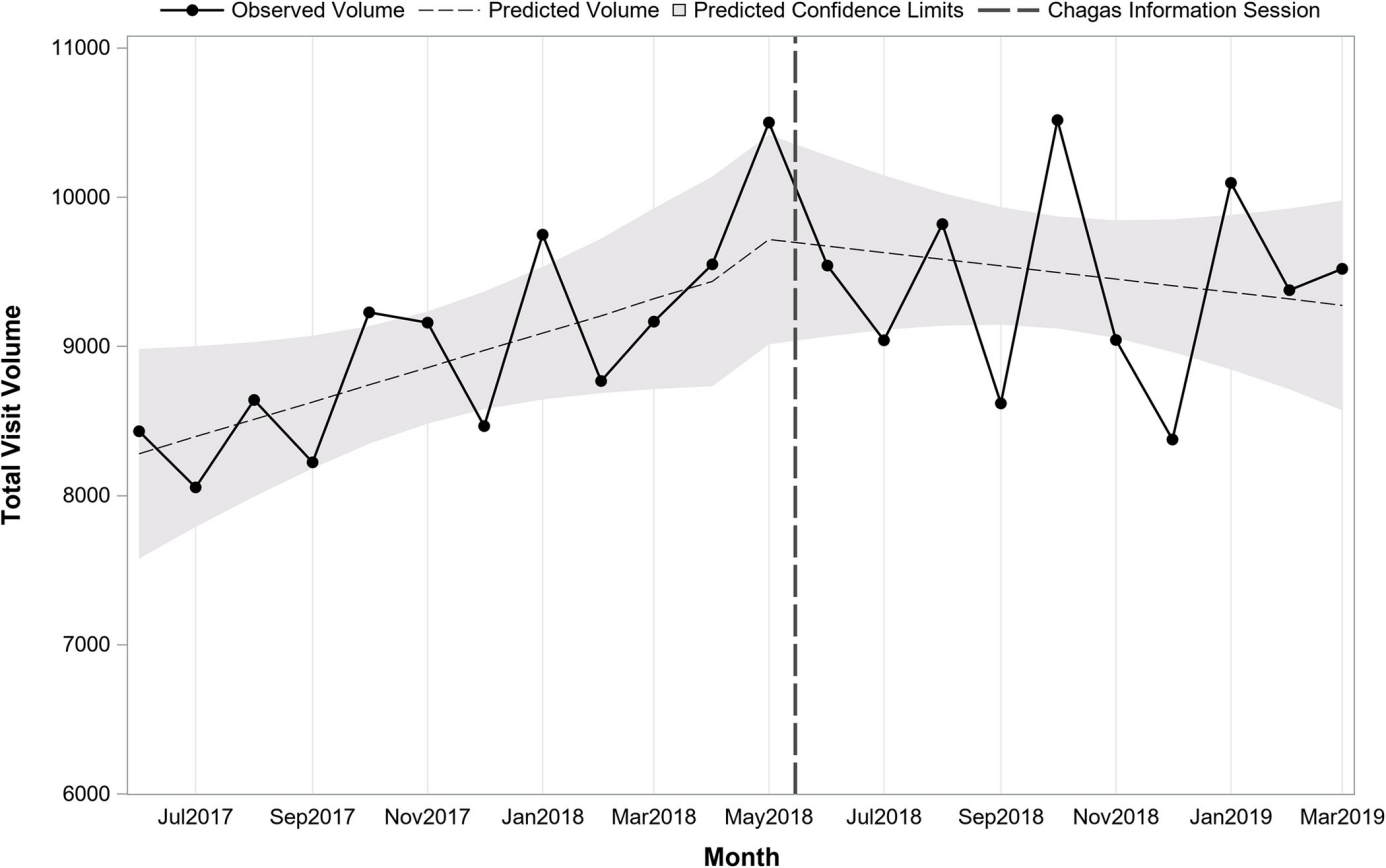
Trend in monthly total patient visit volume before and after Chagas information sessions (N = 201,865 visits; 22 months).

**Fig 8:**
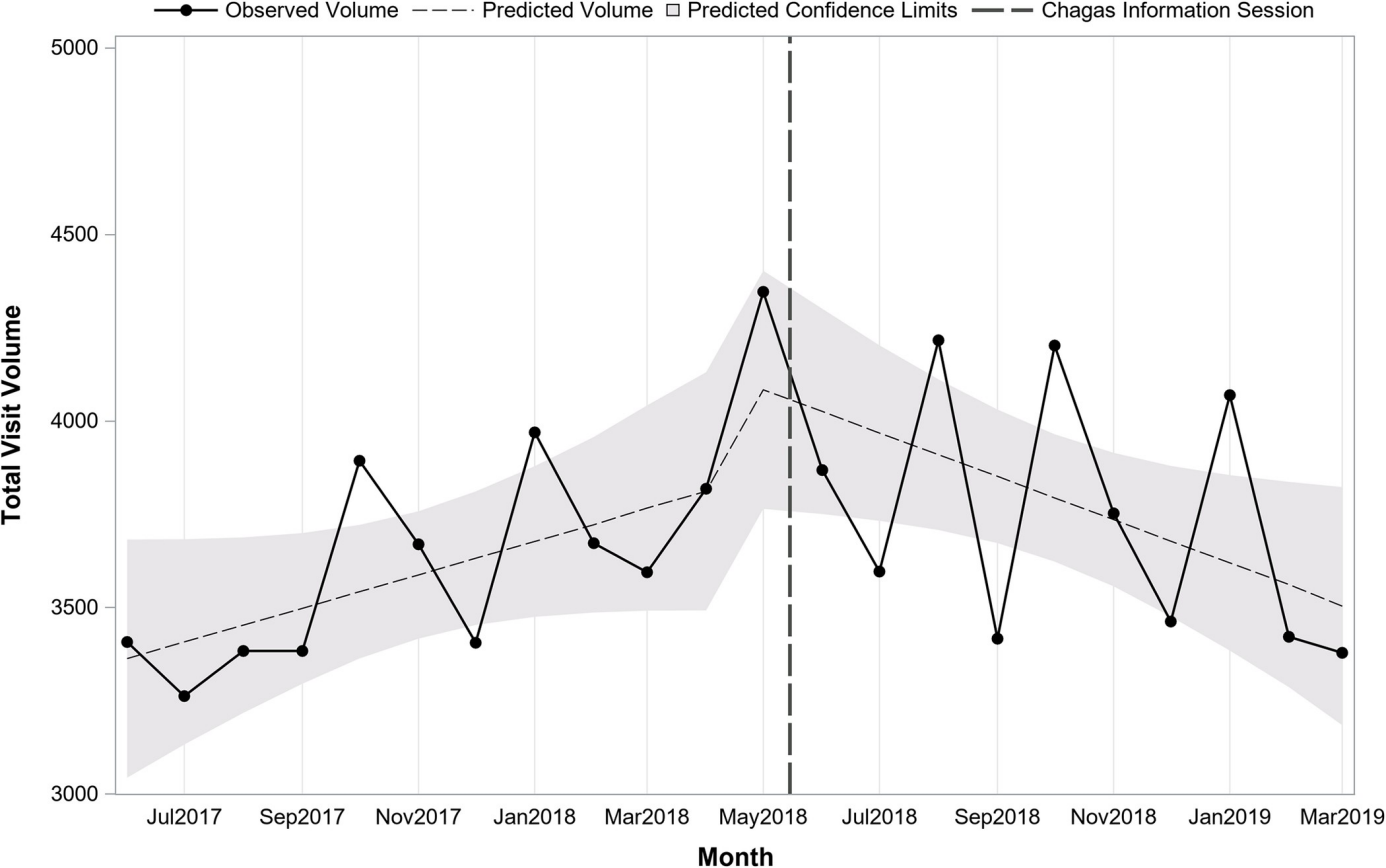
Trend in monthly total patient visit volume before and after Chagas information session in Family Medicine (N = 81,181 visits; 22 months).

**Fig 9:**
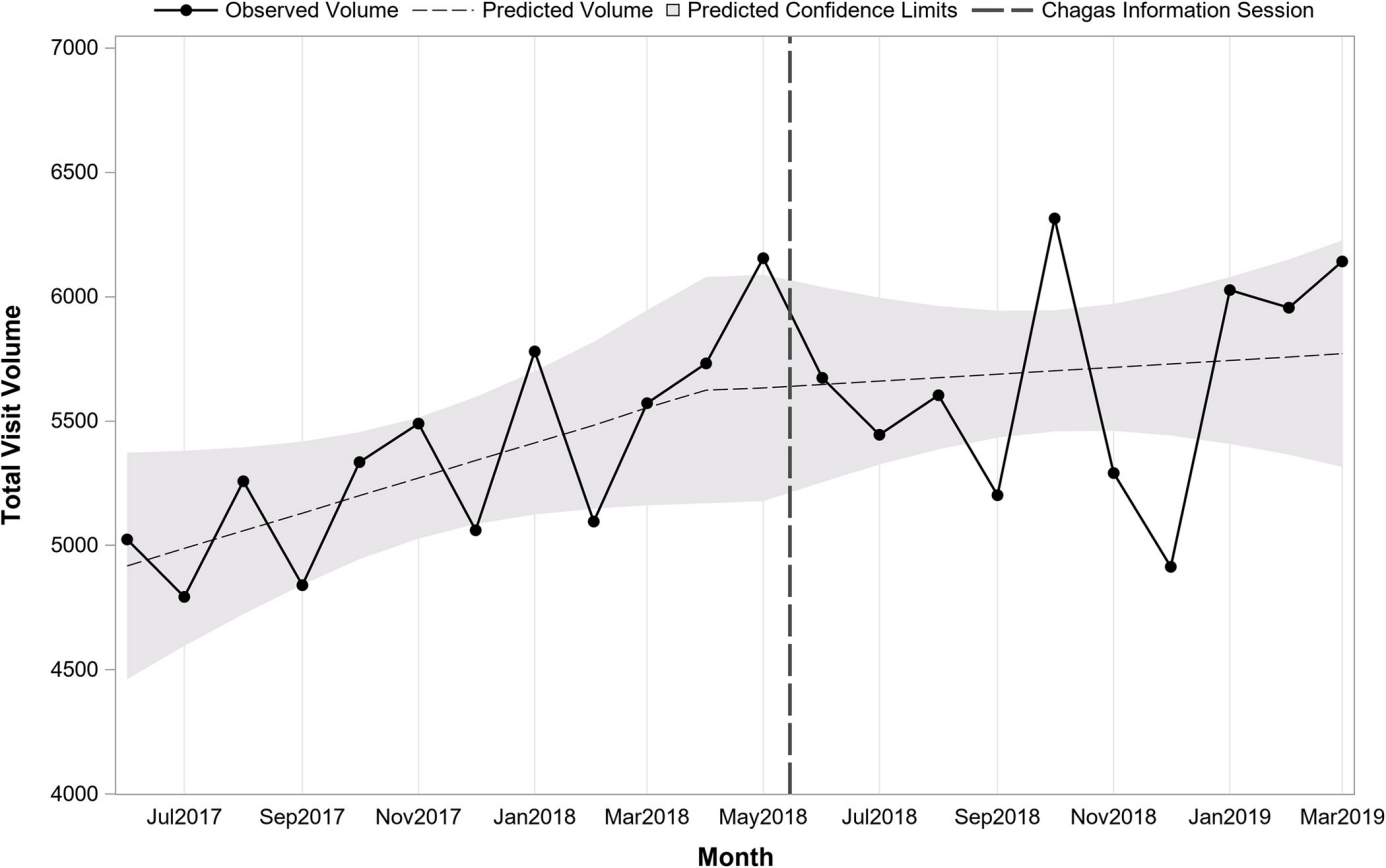
Trend in monthly total patient visit volume before and after Chagas information session in Adult Medicine (N = 120,684 visits; 22 months).

Although we were unable to ascertain the number of at-risk patients seen, we examined the total number of visits within each department and across departments as a balancing measure. Across departments, there were a total of 97,425 visits prior to the sessions vs. 104,440 after. The overall volume across departments indicated no change over time prior to the sessions (pre-session slope = 115.5; p = 0.06), no immediate shift after the sessions (post-session intercept = 325.3; p = 0.53) or shift in slope after the sessions (post-session slope = -159.8; p = 0.06) (Fig 7). For Family Medicine there was no change over time prior to the session (pre-session slope = 44.9; p = 0.10), no immediate shift after the session (post-session intercept = 330.2; p = 0.17), however, there was a change in slope in the post period with a decline over time (post-session slope = -102.9; p = 0.01) (Fig 8). For Adult Medicine there was no change over time before (pre-session slope = 70.7; p = 0.07), no immediate shift (post-session intercept = -4.98; p = 0.99) or change over time after the session (post-session slope = -56.9; p = 0.29) (Fig 9). In summary, when normalized by the number of patient visits, a significant increase in Chagas serology tests sent by both physicians and mid-level providers was noted after two information sessions held on 5/15/2018, one each in the Adult Medicine and Family Medicine departments at EBNHC.

## Discussion

The goal of this analysis was to determine if, after Chagas disease information sessions, testing for *T*. *cruzi* serology increased at a community health center that serves a large Latin American immigrant community. This report focuses on two departments at EBNHC where Chagas disease information sessions were held on May 15, 2018. A total of 4,580 screening tests were performed over the entire study period; 1,957 in the period prior to the information sessions and 2,623 after the sessions. Across departments, testing volume was stable over 10 months prior to the information sessions, increased significantly in the month of the sessions and remained stable over 10 months following the sessions. Findings were similar within departments and by provider type. Before and after periods did not differ with respect to patient’s age, gender, country of origin or Chagas test result.

Following information sessions, testing increased for both physicians and mid-level providers, underscoring the importance of including nurse practitioners, physician assistants and physicians in information sessions. The difference in visit duration and time spent with patients between physicians and mid-level providers may influence the number of tests ordered, however we did not have access to visit duration to explore this relationship. Additionally, the current analysis did not examine whether the prevalence of confirmed Chagas disease cases differed if patients were stratified by age or country of origin. Analysis of these factors will be undertaken in a separate study.

Testing volume after the sessions increased by approximately one-third relative to the period before information sessions. The number of CDC confirmed positive tests also increased by one-third (18 before vs 29 after), however, this difference did not reach statistical significance given the small numbers. In our sample, approximately 0.9% of all screening tests performed resulted in a CDC confirmed positive serology. Given this estimated prevalence, detecting significantly more true positives would require many more tests to be sent or longer periods of observation.

Of the 164 tests sent to the CDC for confirmatory testing 47 (29%) resulted positive, 117 (71%) negative, suggesting low specificity of the Hemagen ELISA. An examination of the number of initial positive, confirmed positive, indeterminate and negative tests performed at EBNHC since 2017 will be reported in a separate analysis. Here we only note that the large fraction of false positive obtained with the Hemagen ELISA placed a large burden on EBNHC staff who had to ensure confirmatory testing at the CDC. We do not know in what way our patients’ test results were affected by the significant variations in sensitivity of Chagas serologic testing recently noted in dependence on patients’ geographic region of origin [28,29]. Truyens and colleagues found that the sensitivity of the ELISA they examined varied between 89.7 and 27.9% [29].

Prior to March 2017 virtually no Chagas diagnostics were performed at EBNHC. In 2016 a single *T*. *cruzi* serology was ordered, compared to nearly 1,600 overall in 2017 as more information sessions were offered and as Strong Hearts volunteers developed systems to follow up test results and to refer patients with confirmed Chagas disease to an Infectious Disease specialty clinic. The system was designed to test every patient from a Latin American country of origin, at the discretion of individual clinicians. We do not know if providers asked patients about specific risks for Chagas diseases, such as familiarity with the insect vector, history of living in houses made from natural materials, risk for congenital transmission, or travel history, though this seems unlikely given the time constraints in a primary care visit. Providers may have sent some tests mistakenly as we found 2% of patients tested originated from the Caribbean and 1% from Africa, though we could not ascertain whether these individuals were at risk for congenital Chagas disease transmission or had travelled to endemic areas in Latin America.

Screening programs facilitate early detection and treatment of disease [30–33]. If untreated an estimated one quarter to one third of patients with *T*. *cruzi* infection will develop non-ischemic cardiomyopathy resulting in death [5]. Establishing diagnostics and treatment of Chagas disease as a standard component of primary care has the potential to reduce morbidity and mortality in low-income Latin American immigrant communities in the US [34,35]. This is especially important since the diagnostic process involves establishing a relationship with the medical system for patients who may not have seen a physician in decades, and management encompasses thorough cardiology follow-up and care.

The complex diagnostic process that requires two testing steps in different laboratories presents significant barriers to Chagas testing [20]. Prior studies have indicated that clinicians agree that Chagas disease screening is important, but often lack the necessary knowledge to make the diagnosis including how to order a test and what to do when a screening test is positive [17–20]. In recent years, the importance of Chagas disease in Latin American immigrant residents of the United States has been highlighted in several publications [6,8,10], yet incorporation of care for Chagas disease into primary care has not occurred systematically across medical institutions in this country. To our knowledge, the East Boston Neighborhood Health Center, part of the “Strong Hearts” Chagas disease project in Boston, Massachusetts, is currently the only medical facility in the US in which Chagas diagnostics are incorporated into primary care in all departments, and referral for treatment and yearly cardiology follow up is standard of care. Goals of the “Strong Hearts” project include filling in Chagas disease knowledge gaps among providers, educating at-risk communities and facilitating the diagnostic, referral, treatment, and follow-up process for Chagas disease patients including supports for social determinants of health [36].

In our information sessions with EBNHC clinicians, we stated that this is a neglected disease of poverty, and that they contribute to ending this neglect whenever they screen a patient and refer a diagnosed patient for tertiary care. In information session updates, we highlighted the results that their work had already obtained. The possibility of making a beneficial impact for a population of patients whom most EBNHC clinicians know well to be harshly discriminated against [37–42] may be an altruistic motivation for them, beyond the benefit to their individual patients. The time periods studied fell before the 2020 renewal of attention on racism in medicine and society [43–46]; current increasing awareness of medical practitioners’ obligation to address racism may facilitate addressing Chagas disease going forward [47–49].

Clinicians who began testing their patients for Chagas disease after learning of the importance of this intervention took on an extra, potentially time-consuming task in their already overly busy workdays. They embraced this further burden purely to improve the health of their patients. We posit that many clinicians are driven by the wish to have tangible, positive impacts on their patients’ lives. The literature on incentives in the healthcare system barely touches on clinicians’ altruism and high professional standards as core motivators [50,51]; yet the adoption of a new diagnostic system by clinicians who received no recognition, personal benefit, or relief from other duties, is strong evidence of the primacy of these motivations for these clinicians. We hypothesize that many clinical staff of community health centers are deeply motivated by the mission to serve their neglected patient populations and to mitigate this neglect.

Quality of clinical care has been experimentally linked to clinicians’ pro-social attitudes [52]. Kolstad and Lindkvist hypothesized that people with “strong pro-social preferences will seek employment in the public health sector rather than in the private for-profit health sector,” [53] and obtained evidence in economics experiments with Tanzanian medical and nursing students in support of this idea [53]. Godager and Wiesen observed heterogeneity in altruism of German medical students: some students in their study attached a higher value to their own profit than to the patient benefit (26%), others (29%) attached equal weights to their own profit and patients’ health benefit while 44% valued patient benefit above their own profit [54]. When personal benefit is measured not only in financial terms, but also in assets like research publications, grants, name recognition or academic advancement, one might expect that heterogeneity between staff who prioritize patients and those who choose personal benefit also impacts allocation of energies of medical facilities’ staff and may affect adoption of Chagas care in a clinical program.

There were no incentives, direct or indirect, given to clinicians for attendance at information sessions or for testing patients. We therefore posit that EBNHC clinicians self-selected to prioritize their patients’ health benefits above resources proceeding to themselves. While generalizability of the findings of Godager and Wiesen is unclear [54], it is notable that a plurality of future clinicians in their study prioritized patient benefit over their personal advantage. Extrapolating to the US medical system, perhaps clinicians working in a federally qualified health center like EBNHC self-select for pro-social preferences and hence are more likely to decide to include another task into their workdays, for the exclusive benefit of their patients. We appealed to clinicians’ sense of justice and to their ability to have a positive systemic impact in our information sessions, and this appeal may resonate particularly with clinician personalities represented at EBNHC.

Our sample is limited to one community health center and hence the findings may not be generalizable to other settings. This was a retrospective analysis and pragmatic evaluation rather than a formal quality improvement project. We were not able to determine the percentage of at-risk patients tested for each time period; however, the overall visit counts were stable or even declining over time in the departments we analyzed. Therefore, an increase in visit volume does not seem to account for our findings. In addition, normalizing tests to the total visit volume each month indicated a similar increase in testing after the information sessions. It is possible that individual patients’ testing requests, word-of-mouth dissemination of Chagas information, as well as individual clinicians seeking out Chagas information and/or CDC recommendations may have influenced the number of tests performed. Additionally, it is not known which clinicians attended the information sessions as attendance is not collected. All clinicians are expected to attend the monthly department meetings, where the information sessions were held. Providers who were not in attendance at the time of the information sessions may have been less likely to send a Chagas test for an at-risk patient. It is possible that fewer tests were performed after the session by Adult Medicine providers if patients seen in the time period studied were previously tested for Chagas disease. As the data was de-identified it is unknown how many patients seen in Adult Medicine between 5/16/2018 and 3/31/2019, the period after the information sessions, were previously tested. In our retrospective study other potential confounding variables (e.g., insurance status, socioeconomic status, and access to care) were not collected and therefore unaccounted for in the analysis. Availability of a basic safety net health insurance for all Massachusetts residents regardless of immigration status is another important, non-generalizable factor influencing our findings.

Importantly, other components of the “Strong Hearts” project are not considered in this analysis. Specifically, community outreach and education are major efforts of the project and have reached thousands of East Boston residents primarily through in-person information in faith-based communities and community-based organizations as well as in English as a Second Language and occupational safety classes, a soup kitchen, health fairs etc. Anecdotally we are aware that this information of community members created a patient “demand” effect that may have motivated some clinicians to test for Chagas disease. Individual clinicians’ motivation to test was not assessed in this study. Strengths of our study are that it supports the effectiveness of one of the four fundamental components of the “Strong Hearts” project, education of medical providers; and that it suggests that altruism and caring are sufficient clinician motivations to enable incorporation of Chagas disease into primary care in the United States.

We believe that diagnostics of Chagas disease can be incorporated into primary care in medical institutions in the United States caring for low-income Latin American immigrants. Providing clinicians with information about the disease is an effective step in the process of establishing systems of Chagas disease care and overcoming the neglect of patients with this disease.

## Conclusion

We conclude that increasing clinician knowledge of Chagas disease is one important step to increase its diagnosis and treatment in at-risk communities. Clinicians may be motivated to take on additional work with no direct benefit to themselves, by knowing they can address a neglected disease among a harshly discriminated population. In addition to clinician education, institutional support, including administrative and laboratory support for diagnosis will be crucial in enabling clinicians to provide appropriate care for the patients with this neglected disease.
